# Flow diversion beyond the circle of Willis: endovascular aneurysm treatment in peripheral cerebral arteries employing a novel low-profile flow diverting stent

**DOI:** 10.1136/neurintsurg-2019-014840

**Published:** 2019-05-14

**Authors:** Stefan Schob, Karl-Titus Hoffmann, Cindy Richter, Pervinder Bhogal, Katharina Köhlert, Uwe Planitzer, Svitlana Ziganshyna, Dirk Lindner, Cordula Scherlach, Ulf Nestler, Jürgen Meixensberger, Ulf Quäschling

**Affiliations:** 1 Department for Neuroradiology, University Hospital Leipzig, Leipzig, Germany; 2 Department of Interventional Neuroradiology, Royal London Hospital, London, UK; 3 Department for Neurosurgery, University Hospital Leipzig, Leipzig, Saxony, Germany; 4 Department for Anesthesiology, University Hospital Leipzig, Leipzig, Germany

**Keywords:** flow diversion, small cerebral vessels, silk vista baby, low profile flow diverter, distal aneurysms

## Abstract

**Background:**

Flow diversion (FD) has emerged as superior minimally invasive therapy for cerebral aneurysms. However, aneurysms of small peripheral vessel segments have not yet been adequately treatable. More specifically, currently established devices necessitate large microcatheters which impede atraumatic maneuvering. The Silk Vista Baby (SVB), a novel flow diverter, offers the as yet unique feature of deliverability via a 0.017 inch microcatheter. This study reports our first experience with the SVB in challenging intracranial vessels employing a vessel-specific tailored microcatheter strategy.

**Materials and methods:**

25 patients (27 aneurysms) were prospectively included. A total of 30 SVBs were employed, predominantly targeting demanding aneurysms of the anterior communicating artery complex. The efficacy of the FD was assessed using two-dimensional vector-based perfusion and conventional digital subtraction angiography (DSA) after implantation and at the first follow-up at 3 months. The first follow-up was available in 22 patients.

**Results:**

All devices were implanted without technical or clinical complications. Eleven treatments were performed using the recommended Headway 17. In 14 interventions the even more maneuverable Excelsior SL10 was used, which was previously tried and tested for safety ’in vitro’ as an alternative delivery system. Aneurysmal influx was strongly reduced after implantation. All parent vessels remained patent. 17/27 aneurysms were completely occluded at first follow-up (∼2.7 months), 6/27 aneurysms showed decreased influx or delayed washout and one remained unchanged. In three cases follow-up DSAs are remaining.

**Conclusions:**

SVB provides enhanced controllability in vulnerable segments beyond the circle of Willis. Smaller variants (2.25 mm and 2.75 mm) can safely be implanted via the superiorly navigable Excelsior SL10. Hence, the SVB represents the next evolutionary step in minimally invasive treatment of cerebral aneurysms.

## Introduction

Flow diversion (FD) has evolved as the endovascular treatment of choice for most types of intracranial aneurysms during the last decade. After its initial employment as a last resort strategy for wide-necked aneurysms,^1^ the field of application has expanded rapidly and nowadays even includes the first treatment for saccular and fusiform-shaped incidental aneurysms located within any of the proximal segments of the circle of Willis,^2–8^ as well as otherwise only inadequately treatable acutely ruptured aneurysms.^9^ The fundamental concept of flow diversion—using devices with distinctly increased surface coverage to redirect blood flow along the physiological axis of the parent vessel, away from the aneurysm sac—aims for incremental remodeling of the affected artery without touching the highly fragile aneurysm itself.^3^ In most cases the implanted flow diverting stent (FDS) gradually induces intra-aneurysmal thrombosis, which—synergistically with the dense mesh of the device itself—provides an ideal matrix for the relatively rapid formation of a neointima that eventually excludes the aneurysm from the circulation.^2^ This approach has proven very successful with high occlusion rates and at least non-inferior complication rates.^8^ Although numerous studies have already demonstrated that FD is an effective and safe therapy for a variety of cerebral aneurysms,^10^ the subgroup of aneurysms originating from distal segments of the circle of Willis has empirically not yet been sufficiently treatable with FDS. More specifically, the delivery systems of currently available well-established FDS such as the Pipeline Embolization Device (PED2, Medtronic, USA), p64 (Phenox, Germany), or SILK (BALT, France) require microcatheters providing large inner diameters (0.021–0.027 inch), which inherently constitute a significant stiffness and thus impede smooth atraumatic maneuverability in the distal circulus segments. As a consequence, only proximal elements of the circle of Willis such as the internal carotid arteries (ICAs), V4 segments, basilar artery, as well as the M1 and A1 segments have been treated regularly with FDS.^3–8^

However, smaller segments of the circle of Willis such as the anterior cerebral artery/anterior communicating artery complex, the middle cerebral artery (MCA) bifurcation, as well as the M2 branches and the pericallosal artery also frequently give rise to especially critical aneurysms with a high risk of rupture.^11^ These segments naturally exhibit two particularly challenging procedural features—namely, the small diameter and distinctly more acute-angled vessel courses. As a consequence, endovascular treatment in these locations remains challenging and novel devices allowing the use of more flexible smaller delivery catheters are warranted.^12–14^

The Silk Vista Baby (SVB) FDS (BALT, France) is a low-profile FDS which achieved CE approval for the use of a 0.017 inch microcatheter in May 2018. This study reports our first experience using the SVB as a low-profile FDS in distal and proximal segments of the circle of Willis.

## Materials and methods

### Ethics approval

Our study, investigating prospectively included cases from June 2018 to December 2018, was approved by the institutional ethics committee (local IRB no AZ 208-15-0010062015). Informed consent of each patient regarding the scientific use of radiological and clinical data was obtained in writing either from the patient or his/her legal representative.

### Patients

We collected cases of elective aneurysm treatments employing solely the SVB FDS as endovascular device. As CE approval for the device was granted in May 2018, we initiated our study in May 2018. Suitable patients who had earlier had an aneurysmal subarachnoid hemorrhage (SAH) and were treated with coiling only in the acute scenario who showed significant aneurysmal relapse and patients with incidentally reported intracranial aneurysms were included in our study. No cases of acute aneurysmal SAH were included. Demographic data, location, size and morphology, immediate clinical and early angiographic follow-up results as well as complications were recorded.

### Interventional procedure

Informed consent for elective endovascular treatment was obtained from all patients. Patients were loaded orally with aspirin 500 mg and ticagrelor 180 mg the day before the procedure. On the day of the intervention our standard regimen was initiated consisting of aspirin 100 mg once a day (indefinitely) and ticagrelor 90 mg twice a day (for 12 months). The efficacy of the antiplatelet regimen was tested in our Department for Laboratory Medicine prior to the intervention in all cases using the Multiplate Analyzer (Roche Diagnostics, Switzerland) and light transmission aggregometry (LTA, Born’s method) as well for confirmation. Both tests were performed to assess the efficacy of platelet inhibition by aspirin (using arachidonic acid as inducer of platelet aggregation) and ticagrelor (using ADP as inducer of platelet aggregation). Sufficient dual platelet inhibition was successfully tested in all cases according to the manufacturers' instructions of the Multiplate system and confirmed by LTA. No case of aspirin or ticagrelor resistance was identified.

All endovascular procedures were performed under general anesthesia using a biplanar angiography suite (Allura Clarity, Philips, The Netherlands). Endovascular access was established via the right femoral artery using an 8F introducer sheath. A bolus of heparin (5000 IE) was administered via the sheath initially. 6F Neuron Max (Penumbra, Alameda, California, USA) was used as the guiding catheter and Sofia 6F was employed as the distal access catheter (Microvention, Aliso Viejo, California, USA) in all cases. The microcatheter was selected after the following considerations.

#### Microcatheter strategy

Different microcatheters, depending on the size of the target vessel, were used for FDS deployment. In cases of proximally located aneurysms affecting segments of rather large diameters (ICA, vertebral artery, M1 segment, A1 segment), the recommended, CE-approved microcatheter Headway 17 (0.017 inch, Microvention Terumo, USA) was successfully used as the FDS delivery system.

After preceding in vitro testing in a flow model (all versions of the 2.25 mm and 2.75 mm device were tested for smooth and uneventful delivery), the Excelsior SL 10 (0.016 inch, Stryker Neurovascular) was used for FDS implantation in small, more peripherally located, significantly curved cerebral arteries (MCA trifurcation, M2–M3 segments, anterior communicating artery, A1–A2 segments, pericallosal artery), as this microcatheter exhibits distinctly enhanced flexibility and allows less forceful and potentially traumatic probing of angiographically demanding vessels. However, this microcatheter was not tried and tested for the CE mark approval, so its application in this regard represents off-label use.

As an important side note, we consider pre-interventional preparation of the Excelsior SL10—especially if 2.75 mm devices are implanted—to be essential. In our experience, friction inside the catheter caused by any FDS during delivery is significantly decreased after the first passage in cases where more than one FDS is necessary to sufficiently treat a large aneurysm. As a consequence of this experience, we initially prepared each Excelsior SL10 using a pREsetT Lite stent retriever (Phenox, Bochum, Germany) in cases of implantation of 2.25 mm and 2.75 mm FDS, which distinctly improved movability/pushability of the FDS through the microcatheter. More specifically, before introducing the flow diverter into the Excelsior SL10, the pREset Lite was carefully deployed in a sterile water basin via the Excelsior SL10 (‘pREsetting’) to reduce intraluminal friction.

However, as ‘pREsetting’ of the SL10 certainly involves an economic problem for the daily routine, we subsequently tested the feasibility of delivery of all 2.25 mm and 2.75 mm devices without prior ‘pREsetting’ in the flow model.

According to our test results, no preparation of the SL10 is necessary if a 2.25 mm device (regardless of its length) is used for implantation. The 2.25 mm diameter devices do not cause a concerning increase in device-related friction when comparing with ‘pREsetting’ to the approach including prior ‘pREsetting’. Therefore, as delivery remains unimpaired, implantation of 2.25 mm variants (although not CE approved) is a recommended strategy for challenging vascular anatomy.

On the other hand, if a 2.75 mm device is intended for use as an endovascular implant, device-microcatheter friction is distinctly increased in cases without prior ‘pREsetting’. As this phenomenon is expected to be aggravated in elongated and more acutely curved vessels, we recommend using either the approved Headway 17 or, if unavoidable, using an accordingly prepared Excelsior SL10.

#### SVB properties and individual selection

The SVB was specifically designed for endovascular-extrasaccular hemodynamic therapy of aneurysms located in small cerebral vessels ranging from 1.5 to 3.5 mm in diameter.^15^ The FDS consists of 48 nitinol wires (ranging from 28 to 45 µm diameter), each designed with a blue oxided surface and an inner platinum core. As nitinol contains roughly 50% nickel, the particular surface modification is essential to prevent release of potentially systemically toxic or locally electrochemically active nickel ions into the circulation^16^ until the device is fully integrated into the neointima. The purpose of the inner platinum core is to enhance the visibility of the complete device, which is especially important to assess impaired opening during the intervention as occurs, for example, due to twisting in curved vessels. The resulting porosity, according to the manufacturer’s information, averages 50–60% with 36–51 pores per mm^2^.

The individual SVB FDS was chosen according to the diameter of the parent vessel. In our study, only devices of 2.25 mm and 2.75 mm diameters were delivered through the Excelsior SL10, requiring the aforementioned small vessel advantages compared with the Headway 17. Technical success (patency of the treated vessel and any covered branches or perforators, flow dynamics, accuracy of FDS placement) was validated angiographically (conventional runs and perfusion imaging) immediately after deployment and again after a waiting period of 15 min.

### Post-interventional course and follow-up

All patients were monitored on an intensive care unit for at least 24 hours post-procedurally. A standardized neurologic examination and a non-enhanced CT of the brain was performed in all cases within 48 hours after the intervention to identify potential hemorrhagic or ischemic complications. Initial angiographic follow-up was scheduled at 1–4 months. The efficiency of FD was assessed at the end of each procedure and at the first follow-up using the O’Kelly–Marotta Scale and the application ‘aneurysm flow’.^17^

## Results

### Patients, implanted devices, and technical feasibility

Twenty-five patients (17 women, eight men) with a total of 27 aneurysms were included in our evaluation. Two of our patients had two distinct aneurysms. One patient had two closely located distal MCA aneurysms (M2–3 segment, patient no 2) which were covered with a single device. The other patient was treated for a relapse of a previously ruptured acutely coiled aneurysm in the proximal M1-segment using a longer device to also cover an incidental ipsilateral ICA aneurysm arising from the orifice of the posterior communicating artery (patient no 17). A total of 30 SVB FDS were implanted. The average patient age was 48 years, ranging from 18 to 70 years. Eighteen patients with a prior episode of acute aneurysmal SAH, treated with coiling or clipping only in the acute stage were included. Fifteen of these cases were treated using the ‘plug and pipe’ strategy. Using this approach, coiling only was performed in acute stage aneurysmal SAH to prevent acute or subacute rebleeding, later followed by flow diversion after a good recovery from SAH.^18^ In three patients the ruptured aneurysms were acutely treated (one clipping, two coiling) and a second incidentally detected aneurysm affecting another segment of the cerebral arteries was later treated preventatively using the SVB FDS. One patient received SVB implantation as complementary treatment for a relapse of a previously stent-assisted coil-treated incidental aneurysm. All of these patients were scheduled for elective reintervention of recurrent aneurysms. The remaining six patients had unruptured aneurysms and were treated primarily with preventatively using the SVB FDS. All aneurysms exhibited sacciform morphology and sizes ranging from 2 to 8 mm. Table 1 shows the demographic and clinically relevant data of the patients.

**Table 1:** Demographic and clinical data of study patients

Patient	Age range	Location	Neck width (mm)	Dome width (mm)	Dome height (mm)	Treatment strategy	Parent artery diameter (mm)	Silk Vista Baby Size	Covered branches	Outcome	DSA follow-up (months)	Aneurysm stasis grade OKM
1	50s	A1–2 left	2	3	2.3	Primary	2	2.25×15	AcomA	Occluded	3	D1
2*	Teenage	M2–M3 right	2	5.6	6.6	Primary	2	2.25×15	Posterior parietal artery	Patent but narrowed	3	D1
2*	Teenage	M2–M3 right	3	3.8	3	Primary	2	2.25×15	Posterior parietal artery	Patent but narrowed	3	D1
3	30s	A1–2 left	3.8	5.5	7.3	Plug and pipe	2.5	2.25×20	Pericallosal artery	Patent	3	D1
4	50s	A2–3 right	1.2	1.7	2.2	Primary	1.8	2.25×10	Pericallosal branch	Patent	4	D1
5	30s	A1–2 left	2	4.5	4.3	Primary	2.2	2.25×10; 2.25×15	AcomA	Occluded	6	D1
6	50s	A1–2 right	2.5	3.6	5.5	Plug and pipe	2	2.25×15	AcomA	Occluded	4	D1
7	40s	PICA left	2.3	3.2	5	Plug and pipe	2.5	2.25×10	PICA	Patent but narrowed	3	D1
8	50s	A1–2 right	2	3.4	4.1	Primary	1.9	2.25×15 (3)	AcomA	Occluded	4	D1
9	40s	A1–2 right	2	4	5	Plug and pipe	2.5	2.25×15	AcomA	Patent but narrowed	3	D1
10	50s	C6 right	4.5	5.8	8.6	Primary	3	3.25×20	Ophthalmic artery	Patent	3	D1
11	70s	PcomA right	3.8	5.4	5.3	Primary	3.3	3.25×25	Ophthalmic artery	Patent	3	A1
12	50s	PICA left	2	5.3	5.8	Plug and pipe	2.8	3.25×10	PICA	Patent	3	B1
13	30s	A1–2 right	2.5	4	5	Plug and pipe	2.5	2.25×15	AcomA	Occluded	3	D1
14	50s	A1–2 left	2	3.3	3.9	Plug and pipe	2.1	2.25×15	AcomA	NA	NA	NA
15	50s	PcomA left	4.1	4.6	3	Primary	3.5	3.25×20; 3.25×25	A1; PcomA	Patent	3	B1
16	60s	A2–3 right	1.8	2.9	3,2	Plug and pipe	1.8	2.25×10	Calloso-marginal artery	NA	NA	NA
17*	30s	PcomA left	3.5	8	11.5	Plug and pipe	2.5	3.25×20	Pcom	Patent	2	B3
17*	30s	M1 left	1.5	2	2.5	Plug and pipe	2.5	3.25×20	A1	Patent	2	D1
18	20s	M1 left	2	4.6	5.1	Plug and pipe	3	3.25×20	A1	Persisting, narrow	1	D1
19	60s	A1–2 left	2	2.5	3.5	Primary	2	2.25×15	AcomA	Persisting, narrow	2	B3
20	50s	C6 left	2.4	3	5.3	Plug and pipe	3.5	3.25×25	Ophthalmic artery	Patent	2	D1
21	40s	PICA left	3.9	3.9	8.3	Plug and pipe	2.6	2.75×25	PICA	Patent	1	D1
22	60s	M1 right	3.5	6	6	Plug and pipe	2.6	2.25×15	Superior trunk	Patent	1	A3
23	50s	A1–2 left	4.9	5.9	4.8	Plug and pipe	2.6	2.75×20	AcomA	Patent	1	B1
24	50s	A1–2 right	3.9	5.3	3.9	Plug and pipe	2.2	2.25×20	AcomA	NA	NA	NA
25	60 s	A1-2 right	2.2	3.3	3.3	Plug and pipe	2.2	2.25×15	AcomA	Occluded	1	D1

*Patients 2 and 17 had two neighboring aneurysms each, which were both treated by implanting only one flow diverting stent per patient

AcomA, anterior communicating artery; DSA, digital subtraction angiography; OKM, O’Kelly-Marotta scale; PICA, posterior inferior cerebellar artery; PcomA, posterior communicating artery.

In brief, 14 treatments were performed in comparatively small vessels exhibiting a challenging morphology in terms of curvature and microcatheter accessibility employing the smaller Excelsior SL10 with higher navigability. Eleven treatments in more accessible comparatively large proximal segments were performed using the Headway 17. For reasons of clarity and comprehensibility, individual applications of FDS and respectively used microcatheters in different segments of the intracranial arteries are summarized in table 2.

**Table 2:** An overview of device and microcatheter distribution with respect to target vessel and aneurysm location in our cohort

Device location	No of treated aneurysms	No of 2.25 mm diameter FDS implanted	No of 2.75 mm diameter FDS implanted	No. of 3.25 mm diameter FDS implanted	Microcatheter: Excelsior SL10	Micro- catheter: Headway 17
Anterior communicating artery complex*	12	14	1	0	11	1
Pericallosal artery	2	2	0	0	2	0
Middle cerebral artery†	5	2	1	1	0	3§
Vertebral artery	3	1	1	1	1	2
Internal carotid –posterior communicating artery‡	5	0	0	6	0	5

* Due to distal shortening in three cases, 15 flow diverting stents (FDS) were implanted.

† One patient had two neighboring middle cerebral artery (MCA) aneurysms which were treated with one FDS (patient no 2).

‡ One case of proximal shortening occurred which demanded implantation of an additional FDS to sufficiently cover the aneurysm.

§One MCA was treated using the same Headway 17 as employed for the treatment of a posterior communicating artery in the last row (patient   no 17).

### Technical success

**Figure 1:**
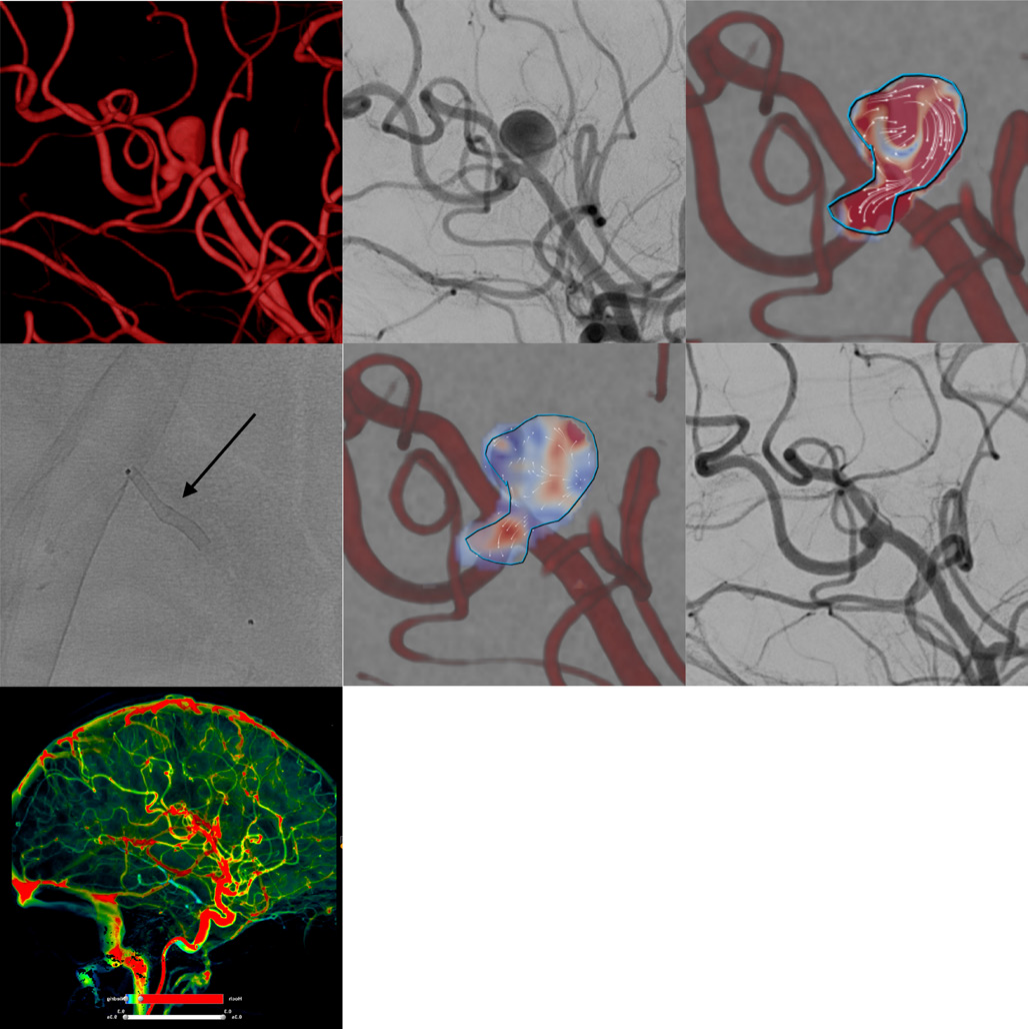
Example of flow diverter stent treatment in the right peripheral middle cerebral artery (M2–3 segment) of a patient with two closely adjacent saccular aneurysms. The upper row (from left to right) shows the three-dimensional angiogram, corresponding conventional digital subtraction angiography (DSA) image in working projection, and intra-aneurysmal flow quantification (employing two-dimensional vector-based imaging), revealing long-lasting turbulent vortical flow in both aneurysmal compartments. The middle row shows the implanted Silk Vista Baby (2.25 mm x 15 mm, arrow), the immediate reduction of aneurysmal influx in vector-based imaging (reduced influx and decreased flow velocity in comparison with the pretreatment image), and the strongly decreased filling of both aneurysms in the conventional DSA image. The bottom row shows maintained normal perfusion of the right hemisphere including the parenchyma from the treated vessels.

**Figure 2:**
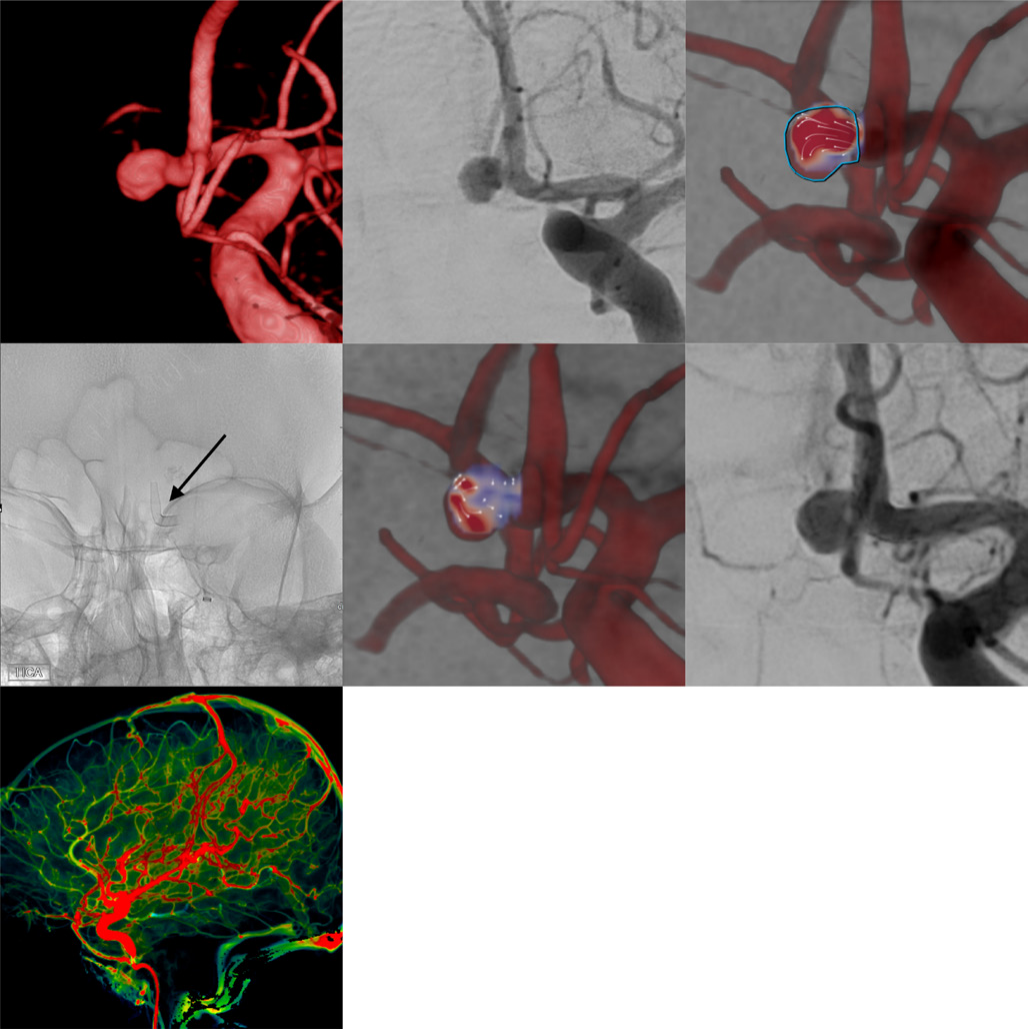
Example of flow diverter stent treatment in the anterior communicating artery complex of a patient with a broad-based saccular aneurysm with predominant supply via the left anterior cerebral artery. The upper row (from left to right) shows the three-dimensional angiogram, the corresponding conventional digital subtraction angiography (DSA) image in working projection, and intra-aneurysmal flow quantification (employing two-dimensional vector-based imaging), revealing turbulent vortical flow in the aneurysmal compartment. The middle row depicts the implanted overlapping Silk Vista Baby stents (2.25×10 mm, 2.25 mm x 15 mm, arrow), the immediate reduction of aneurysmal influx in vector-based imaging (reduced influx and prolonged washout in the aneurysm dome in comparison with the pretreatment image), and the still complete opacification of the aneurysm sac in the conventional DSA image. The bottom row shows maintained normal perfusion of the right hemisphere including the parenchyma from the treated vessels.

**Figure 3:**
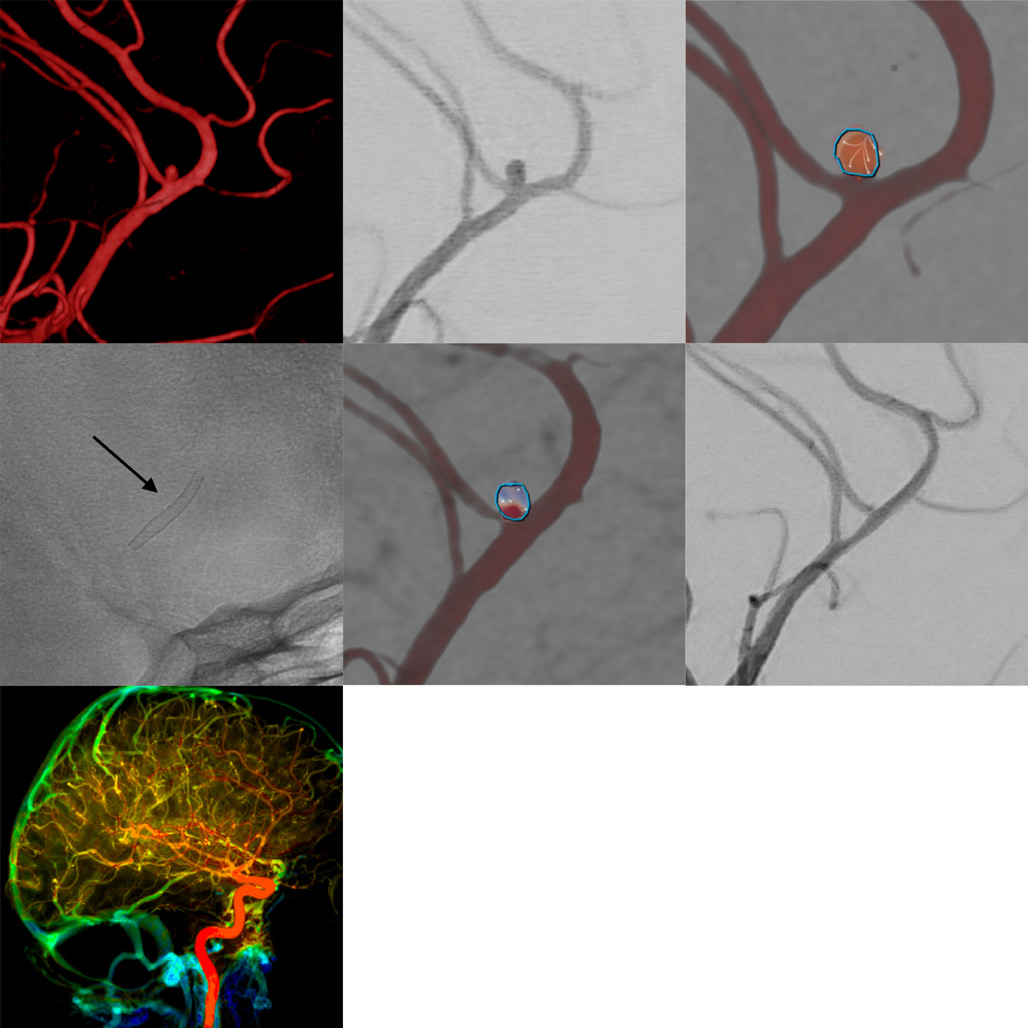
Flow diverter stent implantation for elective hemodynamic treatment of a distal (**A3**) aneurysm in the anterior circulation in a patient with a history of subarachnoid hemorrhage due to a ruptured anterior communicating artery aneurysm. The upper row shows the three-dimensional angiogram, the corresponding conventional digital subtraction angiography (DSA) image in working projection and intra-aneurysmal flow quantification (employing two-dimensional vector-based imaging), revealing short lasting turbulent flow in the aneurysm originating from the proximal pericallosal artery. The middle row shows the implanted Silk Vista Baby stent (2.25×15 mm, arrow), the immediate reduction of aneurysmal influx in vector-based imaging (reduced influx and decreased flow velocity in comparison with the pretreatment image), and the strongly diminished opacification of the aneurysm in the conventional DSA image. The bottom row shows maintained normal perfusion of the hemisphere including the parenchyma from the treated vessels.

As stated above, 30 FDS were implanted for the treatment of 27 aneurysms. All FDS were deployed properly without episodes of proximal or distal non-opening or twisting. Figure 1 provides an example of FDS treatment on the right peripheral MCA (M3 segment) of a patient with two closely adjacent saccular aneurysms. Figure 2 shows an example of FDS implantation in a patient with an incidentally detected anterior communicating artery aneurysm. Figure 3 shows FDS treatment in the distal anterior cerebral artery of a patient with multiple intracranial aneurysms.

### Device shortening

In three cases, distal shortening of less than 5 mm occurred while deploying the respective device in significantly curved segments. As a consequence, to ensure optimal aneurysm coverage, a second longer FDS was implanted additionally in all cases.

In the first case, a 2.25 mm x 10 mm FDS shortened within a strongly curved A1–A2 segment, requiring the supplemental application of a 2.25 mm x 15 mm device. The second case, also caused by distal curve-related shortening in an A1–A2 segment (of a 2.25 mm x 15 mm FDS), required supplemental implantation of an identically sized FDS. The last case occurred in the distal C7 segment; distal curve-related shortening of a 3.25 mm x 20 mm device was compensated by supplemental implantation of a further 3.25 mm x 25 mm device.

In one case, proximal shortening occurred in a 2.25 mm x 15 mm device secondary to undersizing of the FDS in the proximal M1 segment. Due to comparatively large caliber differences in the affected segment, the proximal end of the FDS did not achieve sufficient wall apposition and shortened proximally (approximately 5 mm) shortly after deployment. To compensate for insufficient aneurysmal coverage, a second FDS of greater diameter was applied additionally (2.75 mm x 15 mm). Table 3 provides a synopsis of the cases who experienced shortening.

**Table 3:** An overview of the cases who experienced shortening

Case	Site of implantation	Shortened device	Additional device	Shortening location and extent (mm)
1	A1–A2 left	2.25×10	2.25×15	Distal device 3 mm
2	A1–A2 right	2.25×15	2.25×15	Distal device 3 mm
3	ICA left	3.25×20	3.25×25	Distal device 4 mm
4	MCA left	2.25×15	2.75×15	Proximal device 4 mm

### Immediate flow diverting effect

Direct and indirect signs of the hemodynamic effect caused by FDS implantation were assessed immediately using the O’Kelly–Morotta scale,^17^ aneurysm flow, and two-dimensional angiographic perfusion imaging in standard imaging planes. All but one of the cases did not show an immediate reduction in morphologically assessable intra-aneurysmal flow employing the O’Kelly–Morotta scale (26x A1); one case showed a delayed and reduced inflow in the arterial phase (C1). In all cases, intra-aneurysmal flow velocity was reduced markedly when comparing pre-implantation and post-implantation color-coded vector maps, which were automatically created by the semiquantitative algorithm of the standard application ‘aneurysm flow’ on the Philips console. In the color-coded vector maps, red represents fast aneurysmal flow and blue indicates slow aneurysmal flow. Furthermore, two-dimensional perfusion imaging revealed no significant perfusion deficit in the brain parenchyma of the respective vascular territory. Case examples are shown in figures 1–3.

### Available angiographic follow-up results

First follow-up DSA results are available for 24 aneurysms (22 patients). Eleven complete occlusions (O’Kelly–Morotta: D1) were noted in the first follow-up (after a mean interval of 2.7 months), eight of them within 3 months after the procedure. Three cases of reduced arterial inflow (B1), one case of delayed filling (A3), and two cases of reduced arterial filling with prolonged saccular opacification (B3) were seen. In three patients the first DSA follow-up is not yet available; however, the standardized neurologic examination in the outpatients department 1 month after the procedure revealed no appreciable deficit or disease in those patients.

### Complications

No procedure-related complications were observed in our study. More specifically, no neurologic deficit, intracranial hemorrhage, or ischemic infarction was observable after the procedure or in the first follow-up to date. There was no change in the modified Rankin Scale of any patient in our cohort after the intervention.

## Discussion

Our study provides initial experiences of endovascular aneurysm treatment employing the novel low-profile FDS SVB in proximal and, more importantly, distal segments of the cerebral arteries. Our results underline the procedural safety of the device, especially in the therapeutic context of peripherally located—and hence technically much more demanding—cerebral aneurysms.

Until recently, the treatability of cerebral aneurysms using the well-established endovascular armamentarium (eg, PED2, p64, SILK) was strictly defined by the individual vascular anatomy. More specifically, flow-divertible aneurysms had to be located in easily accessible, mostly proximal segments of the cerebral arteries, as their probeability via the comparatively stiff delivery catheters (0.021–0.027 inch) was inherently limited by increased curvature and small caliber.^12^ In most peripheral aneurysms, even the approach of an exchange maneuver (first probing the target segment with a better navigable microcatheter and then converting to a stiffer delivery catheter via a microguidewire) or other complementary techniques frequently did not result in a sufficient delivery catheter position or the maneuver carried an unjustifiably increased risk of vessel perforation.^19 20^ Therefore, stent-assisted coiling employing low-profile braided stents, which offers better deliverability with a reduced hemodynamic effect and increased risk of procedural perforation due to direct manipulation of the most fragile aneurysm sac, had to be performed in such cases.^21^ Consequentially, great efforts were made to develop FDS for smaller cerebral vessels. However, recently published results employing a smaller next generation FDS offering deliverability via a 0.021 inch catheter demonstrated comparatively high ischemic complication rates while still offering only slightly enhanced navigability.^22^

In our experience, the SVB bridges this significant pitfall of otherwise unparalleled endovascular strategy. The unique feature of the SVB, in comparison with other available FDS^23^, is its intended deliverability via a 0.017 inch microcatheter. As a consequence of this technical advancement, the device provides enhanced navigability and allows comparatively simple access to challenging segments of the intracranial arteries. More precisely, application of the FDS via the Headway 17 for treatment of ‘class 1 elements’ of the circle of Willis (eg, terminal ICAs, A1 and M1 segments, as well as the anterior and posterior communicating arteries) has procedure-wise simplified the intervention significantly. In our study, SVB implantation—even in rather peripheral locations such as the M2–M3 segment—was performed without any difficulties in the 11 cases described above.

However, probing ‘class 2 elements’ of the intracranial circulation (post-communicating and post-bifurcation segments such as A2/A3 and M2/M3) via the Headway 17 empirically still remains risky, especially in acute-angled variants, and sometimes proves to be technically impossible. Therefore, safe deliverability of the smaller SVB variants (2.25 mm and 2.75 mm in diameter) was tested successfully in vitro employing the distinctly more navigable Excelsior SL10 in a flow model. During delivery, the devices with 2.75 mm diameter in particular required increased force to overcome the higher intraluminal friction as a result of the smaller inner diameter compared with the Headway 17. To enhance the pushability of the device through the Excelsior SL10, we first employed a pREset Lite stentriever to smooth the inner surface of the SL10, which resulted in a marked decrease in friction during subsequent FDS delivery. Following this preparative maneuver, delivery of the 2.75 mm SVB FDS via the Excelsior SL10 proved to be equally as safe and feasible as the recommended Headway 17 in our cohort. More precisely, SVB implantation was performed successfully in 14 anatomically demanding cases (11 x AcomA complex, 2 x A3, 1 x VA), without experiencing otherwise common catheter navigability-related difficulties in those small segments. Specifically, in none of the 14 cases did an exchange maneuver have to be performed. Summarizing our experiences with the SL10, devices of 2.25 mm diameter can be implanted uneventfully without prior ‘pREsetting’ whereas the 2.75 mm variants either require the (not CE-approved) preparative maneuver of the SL10 or application of the approved Headway 17.

In our study cohort, three distal shortenings requiring correction by implantation of a second FDS occurred. Two cases were related to the acute-angled A1–A2 transition and one to a bifurcation-adjacent also acute-angled M2 segment, resulting in significant contraction of the distal FDS. Each of the first FDS was chosen following the intention to implant as little as possible intraluminal flow-reducing surface, aiming to reduce the risk of perforator-related ischemic sequelae. Retrospectively, considering the absence of ischemic complications even in cases of overlapping FDS in the A1–A2 transition, it seems appropriate to primarily select longer devices, already discounting for possible device shortening due to curved vascular anatomy.

Furthermore, one case of proximal shortening requiring insertion of a second FDS occurred. This case was related to undersizing of the device, resulting in insufficient proximal wall apposition in an M1 segment exhibiting a large caliber gradient. We therefore conclude that subtle oversizing in vessels showing significant caliber changes is reasonable; however, it should be considered since oversizing may result in lengthening of the FDS.

With respect to the safety and efficacy of aneurysm treatment, the device has shown excellent early results with an aneurysm occlusion rate of almost 70% within an average of 3.3 months after the procedure without signs of apparent ischemic complications. Considering the great visibility and substantial radial force due to the combination of platinum core and nitinol wires, non-opening is probably a less frequently encountered issue than with other devices and, if present, is more easily determinable.

Our study has a number of limitations. First, it only reports early results and thus may not be representative in the long term. Second, the number of patients is small as only approximately 6 months of device availability and a single-center treatment experience are presented.

## Conclusion

Our study demonstrates the peri- and post-procedural safety and efficacy of the novel low-profile SVB FDS for the treatment of proximal and—more importantly—peripheral aneurysms of the cerebral arteries using a 0.017 inch Headway catheter. Furthermore, the potential of the device to treat small cerebral vessels is seemingly greater than initially assumed, as the smaller variants of the FDS (2.25 mm and 2.75 mm) can safely be implanted via the much greater navigable, but not yet CE approved, Excelsior SL10 catheter. In our opinion, the device is setting a new standard for flow diversion in small intracranial vessels.
